# Errors, Omissions, and Offenses in the Health Record of Mental Health Care Patients: Results from a Nationwide Survey in Sweden

**DOI:** 10.2196/47841

**Published:** 2023-11-03

**Authors:** Annika Bärkås, Anna Kharko, Charlotte Blease, Åsa Cajander, Asbjørn Johansen Fagerlund, Isto Huvila, Monika Alise Johansen, Bridget Kane, Sari Kujala, Jonas Moll, Hanife Rexhepi, Isabella Scandurra, Bo Wang, Maria Hägglund

**Affiliations:** 1 Participatory eHealth and Health Data Research Group Department of Women's and Children's Health Uppsala University Uppsala Sweden; 2 MedTech Science & Innovation Centre Uppsala University Hospital Uppsala Sweden; 3 Faculty of Health University of Plymouth Plymouth United Kingdom; 4 Division of General Medicine, Department of Medicine Beth Israel Deaconess Medical Center Harvard Medical School Boston, MA United States; 5 Department of Information Technology Uppsala University Uppsala Sweden; 6 Norwegian Centre for E-Health Research University Hospital of North Norway Tromsø Norway; 7 Department of ALM Uppsala University Uppsala Sweden; 8 Department of Clinical Medicine, Telemedicine and E-health Research Group Arctic University of Norway Tromsø Norway; 9 Business School Karlstad University Karlstad Sweden; 10 Department of Computer Science Aalto University Espoo Finland; 11 Centre for Empirical Research on Information Systems School of Business Örebro University Örebro Sweden; 12 School of Informatics University of Skövde Skövde Sweden

**Keywords:** electronic health records, EHR, mental health, mental health care, national survey, online records access, open notes, ORA, patient-accessible electronic health record, PAEHR, patients, user experiences

## Abstract

**Background:**

Previous research reports that patients with mental health conditions experience benefits, for example, increased empowerment and validation, from reading their patient-accessible electronic health records (PAEHRs). In mental health care (MHC), PAEHRs remain controversial, as health care professionals are concerned that patients may feel worried or offended by the content of the notes. Moreover, existing research has focused on specific mental health diagnoses, excluding the larger PAEHR userbase with experience in MHC.

**Objective:**

The objective of this study is to establish if and how the experiences of patients with and those without MHC differ in using their PAEHRs by (1) comparing patient characteristics and differences in using the national patient portal between the 2 groups and (2) establishing group differences in the prevalence of negative experiences, for example, rates of errors, omissions, and offenses between the 2 groups.

**Methods:**

Our analysis was performed on data from an online patient survey distributed through the Swedish national patient portal as part of our international research project, NORDeHEALTH. The respondents were patient users of the national patient portal 1177, aged 15 years or older, and categorized either as those with MHC experience or with any other health care experience (nonmental health care [non-MHC]). Patient characteristics such as gender, age, education, employment, and health status were gathered. Portal use characteristics included frequency of access, encouragement to read the record, and instances of positive and negative experiences. Negative experiences were further explored through rates of error, omission, and offense. The data were summarized through descriptive statistics. Group differences were analyzed through Pearson chi-square.

**Results:**

Of the total sample (N=12,334), MHC respondents (n=3131) experienced errors (1586/3131, 50.65%, and non-MHC 3311/9203, 35.98%), omissions (1089/3131, 34.78%, and non-MHC 2427/9203, 26.37%) and offenses (1183/3131, 37.78%, and non-MHC 1616/9203, 17.56%) in the electronic health record at a higher rate than non-MHC respondents (n=9203). Respondents reported that the identified error (MHC 795/3131, 50.13%, and non-MHC 1366/9203, 41.26%) and omission (MHC 622/3131, 57.12%, and non-MHC 1329/9203, 54.76%) were “very important,” but most did nothing to correct them (MHC 792/3131, 41.29%, and non-MHC 1838/9203, 42.17%). Most of the respondents identified as women in both groups.

**Conclusions:**

About 1 in 2 MHC patients identified an error in the record, and about 1 in 3 identified an omission, both at a much higher rate than in the non-MHC group. Patients with MHC also felt offended by the content of the notes more commonly (1 in 3 vs 1 in 6). These findings validate some of the worries expressed by health care professionals about providing patients with MHC with PAEHRs and highlight challenges with the documentation quality in the records.

## Introduction

Online patient portals are a common means for patients to gain online record access (ORA), often through services referred to as patient-accessible electronic health records (PAEHRs). Through PAEHRs, patients are offered access to, for example, test and laboratory results, a list of prescribed medications, and referrals. Clinical notes, or narrative visit reports, written by the clinician are considered an essential part of any PAEHR and are often referred to as “open notes” when shared with patients [1]. Online patient portals are becoming more widespread internationally. The European Commission has proposed the European Health Data Space, with one of its aims being to give all European Union citizens access to their electronic health record (EHR) [2]. Since April 2021, the US federal law 21st Century Cures Act has mandated all health organizations to offer patients secure online access to their clinical information housed in their EHRs, including notes written by clinicians, tests, lab results, and referrals; however, access excludes psychotherapy notes [3].

In research, patients with mental health conditions have reported that online access to their mental health records increases feelings of greater validation [4], engagement [4-6], potentially augments patient autonomy [4,7], patient empowerment [7-9], and can increase trust in their clinician [4-6]. Other common experiences perceived by patients with mental health conditions when reading their mental health notes include feelings of having control of their care [4,10-12], and increased understanding of their mental health [10,11], and their medication’s potential side effects [10,11,13,14].

However, albeit limited in numbers, some studies report that some patients with mental health conditions could feel worried or offended by the content of the notes [4,5,10-12]. Patients sometimes find the content of the notes judgmental, disrespectful, and inaccurate [4-6,10] and are worried about being misinterpreted by the health care professional (HCP) and finding errors in the notes [15]. The latter is supported by previous research where experts in a Delphi survey agreed that they expected errors to arise in patients’ mental health notes [16]. Another qualitative survey of stakeholders, including mental health professionals, patients, and informaticians, concluded that further refinement of exemption policies, clinician training, and patient guidance are required in writing mental health notes [17]. Furthermore, due to patient access, mental HCPs note that they might change how they document, such as intentionally leaving out essential clinical information [18-25], concerned that patients might be offended, confused, or anxious by what they read [10,18-21,26]. Some HCPs have reported keeping a “shadow record” due to patient access—in other words, keeping notes outside the official EHR system [27]. HCPs in mental health care (MHC) have also expressed fear of threats or violence from patients and adverse effects on the therapeutic alliance [18,28]. However, some HCPs report benefits with patient access to their mental health notes, such as strengthened patient-provider relationships [22,26,29,30], increased feelings of trust [20,26,30], and increased transparency [21,26,30].

MHC in Sweden encompasses physicians, psychologists, nurses, assistant nurses, occupational and physical therapists, social workers, and medical secretaries from inpatient care, outpatient care, and psychotherapy care. In Sweden, it is possible to receive MHC at the primary care level. Patients have access to their mental health information through *Journalen*, the Swedish national PAEHR, where patients are offered to see their laboratory and test results, diagnoses, referrals, and medications and read their clinical notes from MHC in 17 out of 21 regions [1]. Clinical information is shared with patients in *Journalen* regardless of the health care profession or health care setting. Since Sweden has a decentralized health care system with 21 autonomous regions, there are geographical differences with respect to the mental health information patients can access in the PAEHR, depending on where the patient seeks care [1].

Sharing access to mental health records is still controversial and raises ethical concerns about balancing openness with the risk of harm [31,32]. Many psychiatric organizations and regions in Sweden have resisted the implementation as HCPs worry that patients will become anxious, confused, or upset by what they read [18,20,23]. Like in other countries where the practice is implemented, research on patients with MHCs’ experiences of accessing and reading their mental health records is limited. Previously published studies exploring the experiences of patients with MHC have been conducted predominantly in the United States [15]. A Swedish national patient survey was conducted in 2016 to investigate patients’ experiences accessing their EHR [8], however, with a limited focus on mental health. In the NORDeHEALTH research project [33], an online patient survey was performed to explore the experiences of patients’ ORA [34], providing a greater opportunity to investigate the experiences of patients with MHC with accessing and reading their PAEHR. Importantly, most or all research on the interaction between the presence of mental health diagnoses and experience with PAEHR has focused on patients with specific diagnoses [15,32]. This excludes the wider user population, who may have experience with MHC but not necessarily a formal diagnosis.

This study aims to understand if and how patients’ experiences of ORA differ depending on whether they have received MHC or not. More specifically, we will (1) explore how participant characteristics and interaction with the national PAEHR differ between patients who have received MHC and those who have not, and (2) compare the rate of errors, omissions, and offenses between patients who have received MHC and those who have not.

## Methods

### Overview

To study patients’ experiences of ORA, we conducted an online nationwide survey as part of NORDeHEALTH, our international research project studying the implementation of patient portals in Estonia, Finland, Norway, and Sweden [33]. The survey is known as the NORDeHEALTH 2022 Patient Survey, and its methodology is fully described elsewhere [34]. This paper focuses on the Swedish data set.

### Survey Structure

The whole Swedish survey consisted of 45 questions (38 closed-ended and 7 free-text) divided into seven thematic sections: (1) sociodemographic information; (2) experience with health care; (3) experience with ORA through the patient portal; (4) reasons for and impact of using the health record; (5) errors, omissions, and offenses; (6) security and privacy; and (7) usefulness of information and functions [34]. This study focuses on the following sections: sociodemographic information; experience with health care; experience with ORA through the patient portal; reasons for using the patient portal (subsection: having access to my health record); errors, omissions, and offenses. There were both single- and multiple-choice questions with various response options: “yes” and “no” answers, and Likert scale ratings. All of the included closed-ended questions were mandatory to answer. Questions were partially developed based on previous studies (Moll et al [8], Kujala et al [35], and Zanaboni et al [36]). Before distribution, the survey was pilot-tested with patients. The pilot study proved to be useful in providing insights into the questions, which involved some changes, such as new words and changes to the sequence of the questions. In Sweden, the survey was administered only in Swedish (Multimedia Appendices 1 and 2).

### Data Collection

The survey was distributed through the national patient portal, 1177. The survey link was placed as a notification that appeared to patient users after logging into their national patient portal accounts and entering the PAEHR section of the portal, *Journalen*. This ensured that only verified users of the portal were surveyed. The data collected through the survey were not linked to the patient accounts, so they remained anonymous. The survey was built using Webropol (Webropol Sverige AB) and was available for 3 weeks from January 23, 2022. Only participants aged 15 years or older were eligible to take part. Participation was voluntary. Data were collected through convenience sampling; there were no preset quotas for gender, age, or other sociodemographic characteristics.

### Group Definition

In total, 13,008 patient users answered the online survey. In line with the study’s aims, we focused on a subsample of patients with MHC experience and those with any other health care experience. This was achieved by filtering through the question, “Have you been in contact with a health care professional in the last two years for any of the following?” The answer options were “cancer,” “mental health,” “other health problems,” and “no care or treatment.” It was a multiple-choice question, so participants could select as many as appropriate (Table 1). Participants who indicated no care or treatment (674/13,008, 5.18%) were excluded from the analysis. Those who chose the mental health option were included in the MHC group regardless of whether they also selected the options for cancer and other health problems. Those who did not choose the mental health option and did not select “no care or treatment” were included in the nonmental health care group (non-MHC).

**Table 1:** Group definition.

Survey item		MHC^a^ (n=3131), n (%)	non-MHC^b^ (n=9203), n (%)
**Have you been in contact with a health care provider in the last 2 years for any of the following^c^?**			
	Mental health	3131 (100)	N/A^d^
	Cancer	174 (5.56)	1530 (16.63)
	Other health problems	2441 (77.96)	8605 (93.50*)*
**On what level of care did you receive MHC^c^?**			
	Primary care	1937 (61.87)	N/A
	Hospital outpatient	1809 (57.78)	N/A
	Hospitalized or inpatient	443 (14.15)	N/A
	Emergency care	386 (12.33)	N/A
**Duration of MHC**			
	Less than 3 months	471 (15.04)	N/A
	3 months to 1 year	528 (16.86)	N/A
	1 to 3 years	520 (16.61)	N/A
	Greater than 3 years	1612 (51.49)	N/A
**Have you read about your MHC online in *Journalen*?**			
	Read all or almost all of my records	2105 (67.23)	N/A
	Read some of the records	697 (22.26)	N/A
	Did not read the record	329 (10.51)	N/A

^a^MHC: mental health care.

^b^non-MHC: nonmental health care.

^c^Due to the question being multiple-choice, the total will not add up to 100%.

^d^N/A: not applicable.

### Statistical Analysis

Analysis focused on closed-ended questions from the sections on sociodemographic information; experience with health care; experience with ORA through the patient portal; reasons for using the patient portal (having access to my health record); errors, omissions, and offenses (Multimedia Appendix 1). To address aim 1, we calculated descriptive statistics for participant characteristics (gender, age, education, health care education, employment, and health status) and variables describing portal use experience (frequency of use, encouraged or reminded to read the EHR, and positive and negative experience with the PAEHR). Groups were compared on single-choice questions through the Pearson chi-square test, for which statistical significance was set at *P*<.05. To address aim 2, we calculated summative statistics.

To further analyze group differences in the rates of perceived errors, omissions, and offenses, we used stratified random sampling with proportional allocation [37]. Through it, we created subsamples of the MHC and non-MHC groups that were matched by gender and age. The size of the strata was dictated by the MHC group data (Multimedia Appendix 3). The numbers of participants with “other” as gender response did not match the numbers between the groups for stratification and were thus omitted from both subsamples (n=76; Multimedia Appendix 3). Descriptive statistics and chi-square test were then used to compare the resultant subsamples. The groups were compared on single-choice questions through Pearson chi-square test, for which statistical significance was set at *P*<.05.

All analyses were carried out in JASP (version 0.16.2; University of Amsterdam) by 2 researchers (AB and AK) independently. Figures were created with Datawrapper (Datawrapper GmbH). Free-text responses will be presented in a future publication to allow for an in-depth qualitative analysis. All data were used, and no outliers were eliminated. A second researcher (AK) validated the data presented in the results.

### Ethical Considerations

Ethical approval was granted by the Swedish Ethical Review Authority (EPN 2021/05229) before data collection. Informed consent was acquired at the start of the survey. If participants did not consent to the outlined conditions, they did not proceed with the survey, and no data were recorded. All the collected data were anonymous. Study participants received no compensation.

## Results

### Overview

**Figure 1:**
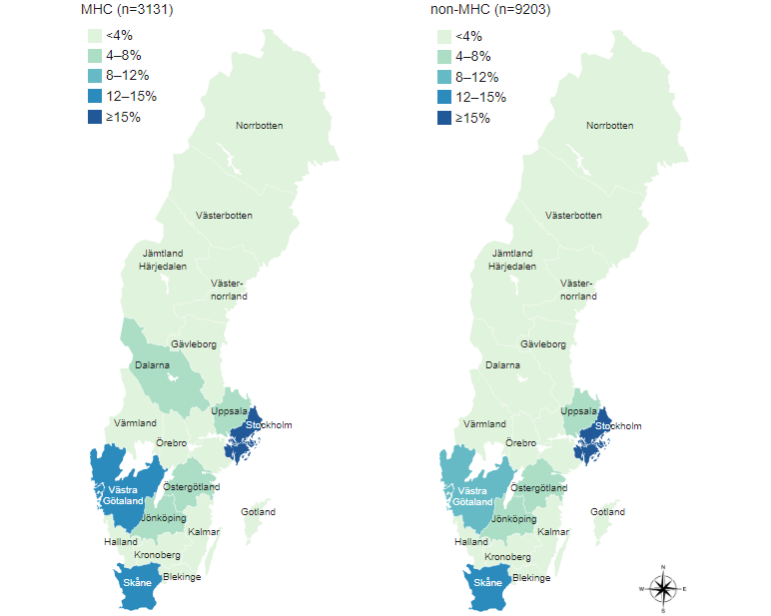
Geographical distribution of which region the respondents’ health care mainly takes place. MHC: mental health care; non-MHC: nonmental health care.

Out of 13,008 participants, a total of 12,334 (94.82%) stated that they had received some care or treatment in the last 2 years. Of these, a quarter (3131/12,334, 25.39%) reported seeking MHC in the last 2 years, and the majority (9203/12,334, 74.61%) indicated they required other health care (non-MHC). The geographical distribution of both groups was comparable, but some differences were noted (Figure 1). There were more MHC respondents in the Dalarna and Västra Götaland regions than respondents from the non-MHC group. In both groups, the highest proportion of responses came from the Stockholm and Skåne regions.

### Participant Characteristics

Both MHC and non-MHC respondents most commonly identified themselves as women (MHC 2373/3131, 75.79%, and non-MHC 5936/9203, 64.5%), but the proportion of men was higher in the non-MHC group (MHC 701/3131, 22.39%, and non-MHC 3248/9203, 35.29%; Table 2). Notably, more participants identified as another gender in the MHC group (MHC 57/3131, 1.82%, and non-MHC 19/9203, 0.21%). The largest age group among the MHC respondents was aged between 25 and 34 years (820/3131, 26.19%), while the largest age group among the non-MHC respondents was aged between 65 and 74 years (2403/9203, 26.11%). Among respondents in the MHC group, the most common education level was upper secondary education (920/3131, 29.38%), while in the non-MHC group, the most common education level was second-cycle higher education (2593/9203, 28.18%). In both groups, most of the respondents had no education in health care. The most common employment status in the MHC group was full-time employment (1156/3131, 36.92%), while in the non-MHC group, most of the respondents were retired (3717/9203, 40.39%), closely followed by full-time employment (3555/9203, 38.63%). In both groups, the overall health among the respondents was “fair” (MHC 1149/3131, 36.70%, and non-MHC 3723/9203, 40.45%), but the proportion of participants with poor health, that is, rated as “bad” or “very bad,” was higher among MHC respondents (1225/3131, 39.12%) than the non-MHC respondents (1920/9203, 20.86%).

**Table 2:** Sample descriptives.

Survey item			MHC^a^ (n=3131), n (%)		non-MHC^b^ (n=9203), n (%)		*P* value^c^	
**Gender**								<.001
	Woman	2373 (75.79)		5936 (64.50)				
	Man	701 (22.39)		3248 (35.29)				
	Other	57 (1.82)		19 (0.21)				
**Age (years)**								<.001
	15-19	120 (3.83)		73 (0.79)				
	20-24	239 (7.63)		153 (1.66)				
	25-34	820 (26.19)		762 (8.28)				
	35-44	656 (20.95)		1053 (11.44)				
	45-54	638 (20.38)		1521 (16.53)				
	55-64	467 (14.92)		1972 (21.43)				
	65-74	141 (4.50)		2403 (26.11)				
	75-84	48 (1.53)		1181 (12.83)				
	>85	2 (0.06)		85 (0.92)				
**Highest attained education**								<.001
	No formal education	19 (0.61)		49 (0.53)				
	Primary school	311 (9.93)		730 (7.93)				
	Upper secondary education	920 (29.38)		2325 (25.26)				
	Higher education: vocational	464 (14.82)		1431 (15.55)				
	Higher education: ≤3 years	584 (18.65)		1769 (19.22)				
	Higher education: >3 years	774 (24.72)		2593 (28.18)				
	Higher education: research (licentiate or PhD)	59 (1.88)		306 (3.33)				
Have health care education			1100 (35.13)		2971 (32.28)		<.001	
**Employment status**								<.001
	Full-time	1156 (36.92)		3555 (38.63)				
	Part-time	431 (13.77)		756 (8.21)				
	Student	380 (12.14)		288 (3.13)				
	Retired	283 (9.04)		3717 (40.39)				
	Unemployed	143 (4.57)		161 (1.75)				
	Not able to work	397 (12.68)		300 (3.26)				
	None of the above	341 (10.89)		426 (4.63)				
**Health status**								<.001
	Very good	108 (3.45)		774 (8.41)				
	Good	525 (16.77)		2606 (28.32)				
	Fair	1149 (36.70)		3723 (40.45)				
	Bad	961 (30.69)		1626 (17.67)				
	Very bad	264 (8.43)		294 (3.19)				
	Do not know or do not want to answer	124 (3.96)		180 (1.96)				

^a^MHC: mental health care.

^b^non-MHC: nonmental health care.

^c^*P* value is derived from the chi-square test comparing the MHC and non-MHC subsamples on a given variable.

### Interaction With the PAEHR Service

There was a statistically significant association between the respondent group and the frequency of reading the EHR (N=12,334; *χ*^2^_3_=176.9; *P*<.001). Almost half of the MHC group respondents had read their EHR more than 20 times in the last 12 months (1425/3131, 45.48%), compared with a third in the non-MHC group (3027/9203, 32.89%; Table 3). In both groups, most of the respondents indicated that nobody encouraged them to read their EHR (N=12,334; MHC 2028/3131, 64.77%, and non-MHC 6305/9203, 68.51%; *χ*^2^_1_=14.9; *P*<.001). A statistically significant association between the respondent group and negative experiences with the EHR was found (N=12,334; *χ*^2^_1_=42.0; *P*<.001). Almost a third of respondents in the MHC group (999/3131, 31.91%) reported having a very negative experience with the EHR, compared with the non-MHC group (2386/9203, 25.93%).

**Figure 2:**
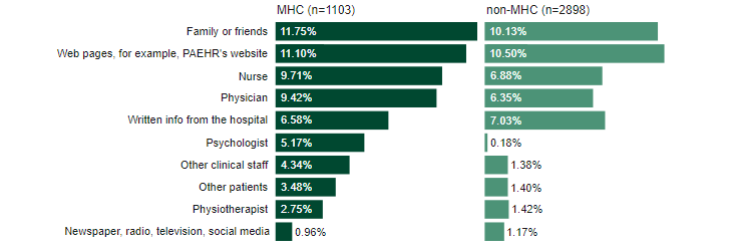
Of those who responded “yes” to the question, “Were you encouraged by someone or reminded to read your EHR?” this figure presents who or what encouraged or reminded them. Due to being a multiple-choice item, the total will not add up to 100%. MHC: mental health care; non-MHC: nonmental health care; PAEHR: patient-accessible electronic health record.

Among participants who noted they had been encouraged by someone or reminded to read their EHR online, most responses were “family and friends” in the MHC group (MHC 368/1103, 11.75%, and non-MHC 932/2898, 10.13%). In the non-MHC group, most responses were “web pages, for example, PAEHR’s website” (MHC 350/1103, 11.10%, and non-MHC 967/2898, 10.50%; Figure 2).

**Table 3:** Interaction with the national patient-accessible electronic health record.

Survey item			MHC^a^ (n=3131), n (%)	non-MHC^b^ (n=9203), n (%)		*P* value^c^
**How often have you read your EHR^d^ during the last 12 months?**						<.001
	This is my first time	56 (1.79)		195 (2.12)		
	2 to 9 times	860 (27.47)		3467 (37.67)		
	10 to 20 times	791 (25.26)		2514 (27.32)		
	Greater than 20 times	1424 (45.48)		3027 (32.89)		
**Were you encouraged by someone or reminded to read your EHR?**						<.001
	Yes	1103 (35.23)		2898 (31.49)		
	No, nobody encouraged me	2028 (64.77)		6305 (68.51)		
Had a very positive experience with the health record			1309 (41.81)	4075 (44.28)		.02
Had a very negative experience with the health record			999 (31.91)	2386 (25.93)		<.001

^a^MHC: mental health care.

^b^non-MHC: nonmental health care.

^c^*P* value is derived from the chi-square test comparing the MHC and non-MHC subsamples on a given variable.

^d^EHR: electronic health record.

**Figure 3:**
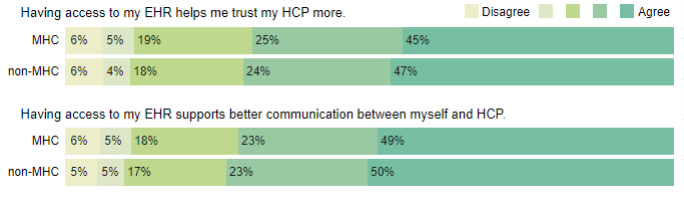
Response distribution to the questions of how access to the electronic health record (EHR) affects trust and communication with health care professionals (HCPs). MHC: mental health care; non-MHC: nonmental health care.

The differences between the groups on how access to EHR improves trust and communication with HCPs were not statistically significant (trust: N=12,334; *χ*^2^_4_=6.8; *P*<.15; and communication: N=12,334; *χ*^2^_4_=5.5; *P*<.24). Respondents in both the MHC group and non-MHC group agreed that trust and communication increased as a result of access to their EHR (Figure 3).

### Errors, Omissions, and Offenses

Errors, omissions, and offensive content were perceived to be more common for the MHC group than the non-MHC group (Table 4). Half of the respondents in the MHC group (1586/3131, 50.65%) reported finding an error in their EHR, and about a third (1089/3131, 34.78%) reported an omission. There was a statistically significant association between the respondent group and identified errors and omissions (errors: N=12,334; *χ*^2^_2_=303.7; *P*<.001, and omissions: N=12,334; *χ*^2^_2_=192.5; *P*<.001), indicating that the MHC respondents perceived more errors and omissions than the non-MHC respondents.

Half of the MHC group (795/1586, 50.13%) rated the most serious error they found as “very important,” and nearly 6 in 10 rated the most serious omission as “very serious” (622/1089, 57.12%). The association between the respondent group and the error rating was significant (N=4897; *χ*^2^_3_=51.5; *P*<.001). Participants in the MHC group rated the errors as more important than those in the non-MHC group. No significant differences were found between the groups regarding the rating of the omissions (N=3516; *χ*^2^_3_=2.5; *P*=.47). In both groups, a large proportion of the respondents reported doing nothing when they found an error or omission (MHC 792/1918, 41.29%, and non-MHC 1838/4359, 42.17%).

The majority of respondents indicated that they had not been offended by something they read. However, the proportion of those who were offended was larger among the MHC respondents: more than a third of the MHC group (1183/3131, 37.78%) reported feeling offended by something they read in their EHR. There was a significant association between the respondent group and offense (N=12,334; *χ*^2^_1_=544.7; *P*<.001).

**Figure 4:**
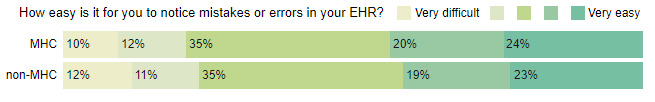
Response distribution to the question of how easy or difficult it is to notice mistakes or errors in the electronic health record (EHR). MHC: mental health care; non-MHC: nonmental health care.

There were small but statistically significant differences between the groups with respect to the ease with which they noticed mistakes or errors in the EHR (MHC 751/3131, 24%, and non-MHC 2117/9203, 23%; Figure 4; N=12,334; *χ*^2^_4_=14.4; *P*<.001).

**Table 4:** Rates of errors, omissions, and offenses.

Survey item					MHC^a^ (n=3131), n (%)		non-MHC^b^ (n=9203), n (%)			*P* value^c^	
**Errors**											
	**Have you found anything that was wrong in your EHR^d^ (not misspellings/typographical)?**									<.001	
		Yes	1586 (50.65)			3311 (35.98)					
		No	904 (28.87)			4253 (46.21)					
		Do not know or do not remember	641 (20.47)			1639 (17.81)					
	**If yes, how important was the worst mistake for you^e^?**									<.001	
		Not at all important	148 (9.33)			508 (15.34)					
		Somewhat important	586 (36.95)			1332 (40.23)					
		Very important	795 (50.13)			1366 (41.26)					
		Not sure	57 (3.59)			105 (3.17)					
**Omissions**											
	**Have you found anything you thought was missing from your EHR?**									<.001	
		Yes	1089 (34.78)			2427 (26.37)					
		No	1059 (33.82)			4418 (48.01)					
		Do not know or do not remember	983 (31.40)			2358 (25.62)					
	**If yes, how serious was the most important missing information for you^f^?**									.47	
		Not at all serious	20 (1.84)			46 (1.90)					
		Somewhat serious	372 (34.16)			895 (36.88)					
		Very serious	622 (57.12)			1329 (54.76)					
		Not sure	75 (6.89)			157 (6.47)					
	**Did you do any of the following when you found a mistake or missing information in your EHR^g^?**									.08	
		Informed health care professional at the next visit	499 (26.02)			1010 (23.17)					
		Contacted care unit by phone	357 (18.61)			837 (19.20)					
		Did nothing	792 (41.29)			1838 (42.17)					
		Something else	270 (14.08)			674 (15.46)					
**Offense**											
	**Have you ever felt offended by something you read?**								<.001		
		Yes	1183 (37.78)			1616 (17.56)					
		No	1948 (62.22)			7587 (82.44)					

^a^MHC: mental health care.

^b^non-MHC: nonmental health care.

^c^*P* value is derived from the chi-square test comparing the MHC and non-MHC subsamples on a given variable.

^d^EHR: electronic health record.

^e^Due to the question being responded to by the “yes” of errors, the total of numbers is 1586 (MHC) and 3311 (non-MHC).

^f^Due to the question being responded to by the “yes” of omissions, the total of numbers is 1089 (MHC) and 2427 (non-MHC).

^g^Due to the question being responded to by the “yes” of errors or omissions, the total of numbers is 1918 (MHC) and 4359 (non-MHC).

### Comparison of Error, Omission, and Offense Rates Between Stratified MHC and Non-MHC Subsamples

To further investigate the rate of errors, omissions, and offenses, we used stratified random sampling with proportional allocation, which resulted in gender- and age-matched MHC and non-MHC subsamples (Table 5). Despite matching the distribution of age and gender, the group differences in rates persisted. Errors were significantly more common in the MHC subsample (errors: N=5830; *χ*^2^_2_=131.4; *P*<.001; as well as omissions: N=5830; *χ*^2^_2_=69.2; *P*<.001; and offenses: N=5830; *χ*^2^_1_=177.7; *P*<.001).

**Table 5:** Rates of errors, omissions, and offenses in the stratified subsamples.

Survey item				MHC^a^ (n=2915), n (%)	Matched non-MHC^b^ (n=2915), n (%)		*P* value^c^	
**Participant characteristics**								
	**Gender**							>.99
		Woman	2254 (77.32)		2254 (77.32)			
		Man	661 (22.68)		661 (22.68)			
	**Age** **(years)**							>.99
		15-19	72 (2.47)		72 (2.47)			
		20-24	147 (5.04)		147 (5.04)			
		25-34	759 (26.04)		759 (26.04)			
		35-44	647 (22.20)		647 (22.20)			
		45-54	634 (21.75)		634 (21.75)			
		55-64	465 (15.95)		465 (15.95)			
		65-74	141 (4.84)		141 (4.84)			
		75-84	48 (1.65)		48 (1.65)			
		>85	2 (0.07)		2 (0.07)			
**Errors, omissions, and offenses**								
	**Error rate**							<.001
		Yes	1471 (50.46)		1067 (36.60)			
		No	858 (29.43)		1230 (42.20)			
		Do not know or do not remember	586 (20.10)		618 (21.20)			
	**Omission rate**							<.001
		Yes	1017 (34.89)		829 (28.44)			
		No	1000 (34.31)		1309 (44.91)			
		Do not know or do not remember	898 (30.81)		777 (26.66)			
	**Offense rate**							<.001
		Yes	1084 (37.19)		621 (21.30)			
		No	1831 (62.81)		2294 (78.70)			

^a^MHC: mental health care.

^b^non-MHC: nonmental health care.

^c^*P* value is derived from the chi-square test comparing the MHC and non-MHC subsamples on a given variable.

## Discussion

### Overview

This study presents the first large study of the perceptions of patients with MHC with errors, omissions, and offenses after accessing their online records in Sweden. The study forms part of a larger data set from the NORDeHEALTH 2022 Patient Survey [34]. A central finding was that 1 in 2 patients with MHC have found errors in their health records (50.65%), and about 1 in 3 have identified missing information (34.78%), with about half of the respondents rating both the worst errors and omissions as “very important.” This is a marked increase from the other patients, of whom one-third identified an error (35.98%) or omission (26.37%), a rate that is already higher than one may consider acceptable. Interestingly, almost half of both patients with MHC (41.29%) and non-MHC (42.17%) chose not to do anything to correct the error or omission. When it comes to rates of offensive content in the EHR, about 1 in 3 patients with MHC have felt offended by their notes (41.29%), compared with 1 in 6 patients with non-MHC (17.56%). Noting that the MHC group included more women as well as younger patient users than the non-MHC group, we created stratified subsamples to investigate whether the group differences in error, omission, and offense rates persisted. We found that the non-MHC subsample reported fewer instances of errors, omissions, and offenses, even when the samples were matched by the distribution of age and gender. Notably, when it comes to the positive impact of EHRs on trust in HCPs and communication with HCPs, both patients with MHC and non-MHC had similarly high levels of agreement with both statements. Both patient groups also had more positive experiences than negative ones.

There could be several reasons why respondents in the MHC group reported more errors and considered them more important than in the non-MHC group. One possibility is that individuals in MHC may have more complex health conditions [38] that require more careful monitoring and documentation, making errors more salient. Additionally, mental health conditions may be more subjective and difficult to quantify, leading to more disagreements or discrepancies in how they are recorded in the EHR. Another possibility is that individuals in MHC may be more attuned to their health and more likely to notice and report errors, while individuals in other health care may be more accustomed to their good health and less likely to scrutinize their EHR for errors. While this difference may be interpreted as a reason for not having open mental health notes or keeping shadow records, neither closed notes nor shadow records are a solution. In a Delphi study [16], there was a consensus among experts that open mental health notes would be helpful for patients to find errors in their notes and be able to correct them. Furthermore, the experts agreed upon the adverse effects of closed notes, such as greater patient stigmatization and harm. Other studies have noted the desire of patients with MHC to access and read their mental health notes to ensure the accuracy of the content and that no errors occur [15].

A possible reason for experiencing more errors in the MHC group could be the same as for the more frequently experienced omissions: the complex medical backgrounds, comorbidities, and medications [38], which may render it more challenging for HCPs to document everything accurately. Additionally, patients with mental health issues may be more likely to have communication difficulties [39] and may be subject to different cultural perceptions between patients and therapists [40], which could result in important information not being recorded in their health records. Given that the overwhelming majority of respondents deemed the missing information important, it suggests that patients recognize the importance of accurate and complete health records. The MHC group experienced something in the notes as offensive at a much higher rate than respondents in the non-MHC group. It is not clear what led to higher offense rates in the MHC group. The posed question did not specify whether it was concerning MHC.

It is unclear why most chose to do nothing when they found an error or omission that they deemed very important. One reason may be that patients fear being perceived as complaining or bothersome if they ask the HCP to correct their health record. These findings do not appear to be in line with research reporting on the fear of HCPs regarding increased workload as a consequence of patients asking for the notes to be explained or corrected [15]. Blease et al [41] discuss the phenomenon of epistemic injustice, as it could arise when an individual has a lower level of credibility due to belonging to a stereotypical group. In this case, patients within MHC already have forms of discretion or negative stereotyping, which further impedes their agency in raising queries or asking for corrections to their records. Correcting the health record is burdensome in more practical terms, as no digital solution exists in the Swedish PAEHR.

In previous research, mental HCPs have noted whether sharing notes should be on a case-by-case basis depending on the treatment or diagnosis and whether all details and information should be included. For example, when treating traumatic experiences, HCPs have recommended excluding detailed information from the notes due to privacy and safety reasons for the patient, as well as the severity of the illness and psychiatric diagnosis [15]. The fear among HCPs of misunderstandings, misinterpretations, and patients finding errors and requesting changes has led to them being less candid, less detailed, and changing the tone of their notes [15]. In Norway, HCPs in psychiatric care have been reported to keep a “shadow record” outside the official EHR system due to patient access [27]. A theory of the MHC respondents’ more significant experiences of omissions could be a case of HCPs adjusting the notes, such as intentionally leaving essential details and information out and altering the tone of the notes, concerned with their beliefs about patients’ increased sensitivity. What has been argued for in previous research [42] is the need for more sustained efforts in terms of continuing medical education for HCPs on how to write notes that patients have access to and can read—especially needed within MHC to strengthen HCPs’ knowledge on how to document notes that patients will read to minimize the risks of patients’ negative experiences of open MHC notes.

An observation of the participant characteristics is that the respondents in the MHC group are generally younger than those in the non-MHC group. MHC respondents peak at ages 25 and 54 years, and the same peak can be found at ages 55 and 74 years in the non-MHC group; however, the most common age range is between 25 and 34 years in the MHC group and between 65 and 74 years in the non-MHC group. In both groups, most respondents have stated they have “fair” health; however, respondents in the MHC group have reported either “bad” or “very bad” health status in a higher proportion than in the non-MHC group. This could suggest that mental health issues are more prominent in young people, which is supported by the fact that respondents in the MHC group have reported either “bad” or “very bad” health status in a higher proportion than in the non-MHC group. In the Västra Götaland and Dalarna regions, there were more MHC respondents than non-MHC respondents. This discrepancy may be due to the differences in what health information patients can access in the PAEHR depending on which region they seek care in [1].

Moreover, a higher proportion of the MHC respondents have had a “very negative” experience with the EHR compared with non-MHC respondents. The sensitive nature of mental health issues could explain this. It is possible that reading about mental health problems could trigger negative emotions in patients, leading to a more negative experience with the EHR. Generally, respondents in the MHC group read their EHR more frequently than in the non-MHC group. One possible explanation is that patients with MHC may need to review their medical records more often to keep track of their treatment progress or to better understand their mental health condition. Additionally, patients with MHC may be more engaged in their health care and more motivated to take an active role in their treatment, which could lead to a more frequent review of their EHR. Finally, it is possible that the higher frequency of EHR review in the MHC group could be due to the younger age of the respondents, who may be more comfortable and familiar with digital technology and therefore more likely to access their EHR. With these inherent group differences, it is perhaps unsurprising that there is a divergence when comparing reported error, omission, and offense rates.

### Limitations and Future Directions

This study had several limitations. The survey relied on self-report, which limits the accuracy of the findings due to response biases. Further, patients with MHC experience were identified based on whether they had been in contact with a HCP with regard to a mental health condition in the last 2 years. This may have been interpreted in various ways by the respondents. Notably, while the study was advertised inside the patient record, the survey and the record were not connected. It was not possible to verify a respondent’s health care history, unlike in a previous US study where patients’ mental health diagnoses were extracted from the patient record [13]. Nonetheless, our follow-up questions on the mental health care experience allowed us to better categorize the patients, for example, on levels of care and length of treatment.

Another limitation stems from the formulation of the gender question. While patients with MHC selected the answer option “other,” it is not clear what their preferred gender label is. Previous research has found that transgender patients have noted experiences of harm when reading their health records [43]. Here, it is possible that part of the increased rate of errors or offensive content is related to gender. In the future, studies should adopt a more inclusive gender question to ensure that gender-sensitive analyses are possible.

In the NORDeHEALTH project, future analyses will focus on the respondents who responded that the level of MHC has been within emergency care and those who reported to have been hospitalized, as they might have experienced serious mental illness.

### Conclusions

In this study, we seek to understand if and how the experiences of patients with and without MHC differ when using PAEHRs. This study reports findings from the largest investigation of the experiences of patients with MHC with accessing online records in Sweden. The analysis revealed that 1 in 2 patients with MHC identified errors in their records, with 1 in 3 identifying omissions or feeling offended by the content of the notes. Moreover, patients in MHC reported these experiences at a much higher rate than other patients. Adding to these concerning findings, most patients reported that they did not act when they found errors or omissions in their records, despite rating them as “very important.”

Additionally, the study raises questions about why respondents in the MHC group detected more missing information than those in the non-MHC group and why they experienced higher offense rates. One reason for more omissions could be that HCPs might adjust the notes to intentionally leave essential details and information out, concerned with their beliefs about patients’ increased sensitivity, highlighting the need for continuous medical education for HCPs on documenting notes that patients will read. Overall, this study highlights identified challenges with the documentation quality in the records. There is a need for better training in how HCPs write notes and the language they use, and for HCPs to recognize that patients can and should act as collaborators with them in improving the records’ accuracy [44].

## Appendix

Multimedia Appendix 1 Survey overview of the items analysed in this study.

Multimedia Appendix 2 Checklist for Reporting Results of Internet E-Surveys (CHERRIES).

Multimedia Appendix 3 Strata of the stratified random sampling.

## Abbreviations

EHR: electronic health record
HCP: health care professional
MHC: mental health care
non-MHC: nonmental health care
ORA: online record access
PAEHR: patient-accessible electronic health record

## Data Availability

The data may be made available upon reasonable request and after consent from the data holder (Maria Hägglund, maria.hagglund@kbh.uu.se).
