# Supplementary material for: Errors, Omissions, and Offenses in the Health Record of Mental Health Care Patients: Results from a Nationwide Survey in Sweden
Source: J Med Internet Res. 2023 Nov 3;25:e47841. doi: 10.2196/47841 (PMC10656659; doi:10.2196/47841)
Supplement: Multimedia Appendix 1 [file jmir_v25i1e47841_app1.docx]

**Multimedia Appendix 1**. Survey overview of the items analyzed in this study. All of the items were mandatory to answer. Note that this overview does not represent the full questionnaire of the NORDeHEALTH 2022 Patient Survey (JMIR Preprints #47573). Left column: items in Swedish, right column: translated items in English.

| **SOCIO-DEMOGRAPHIC INFORMATION** | | | |
| --- | --- | --- | --- |
| Ålder: | | Age: | |
| - 14 eller yngre | | - 14 or younger | |
| - 15 – 19 år | | - 15 – 19 years | |
| - 20 – 24 år | | - 20 – 24 years | |
| - 25 – 34 år | | - 25 – 34 years | |
| - 35 – 44 år | | - 35 – 44 years | |
| - 45 – 54 år | | - 45 – 54 years | |
| - 55 – 64 år | | - 55 – 64 years | |
| - 65 – 74 år | | - 65 – 74 years | |
| - 75 – 84 år | | - 75 – 84 years | |
| - 85 år eller äldre | | - 85 years or more | |
| Kön: | | Gender: | |
| - Kvinna | | - Woman | |
| - Man | | - Man | |
| - Annat | | - Other | |
| Vilken är din högst avslutade utbildning? | | What is your highest attained education? | |
| - Ingen formell utbildning | | - No formal education | |
| - Grundskola eller motsvarande | | - Primary school | |
| - Gymnasieexamen eller motsvarande | | - Upper secondary education | |
| - Eftergymnasial utbildning, ej högskola/universitet | | - Higher education: vocational | |
| - Högskole-/universitetsutbildning, 3 år eller mindre | | - Higher education: 3 years or less | |
| - Högskole-/universitetsutbildning, mer än 3 år | | - Higher education: more than 3 years | |
| - Utbildning på forskarnivå (doktor, licentiat) | | - Higher education: research (PhD or licentiate) | |
| Har du utbildning inom vården? | | Do you have a health professional education? | |
| - Ja | | - Yes | |
| - Nej | | - No | |
| Vilket av följande beskriver bäst din sysselsättning för närvarande? Välj det svar som passar dig bäst. | | Which of the following best describes your current employment status? Choose the one that is most relevant. | |
| - Heltidsarbete | | - Full-time | |
| - Deltidsarbete | | - Part-time | |
| - Student | | - Student | |
| - Pensionär | | - Retired | |
| - Arbetslös | | - Unemployed | |
| - Kan inte arbeta | | - Not able to work | |
| - Inget av ovan | | - None of the above | |
| Region där din vård huvudsakligen sker: / Region where your care mainly takes place: | | | |
| - Blekinge | - Jönköpings län | - Sörmland | - Västra Götaland |
| - Dalarna | - Kalmar län | - Uppsala län | - Örebro län |
| - Gotland | - Kronoberg | - Värmland | - Östergötland |
| - Gävleborg | - Norrbotten | - Västerbotten | - Vill inte uppge/Do not want to state |
| - Halland | - Skåne | - Västernorrland |  |
| - Jämtland Härjedalen | - Stockholms län | - Västmanland |  |
| **EXPERIENCE WITH HEALTHCARE** | | | |
| Hur är din allmänna hälsa? | | What is your overall health? | |
| - Väldigt bra | | - Very good | |
| - Bra | | - Good | |
| - Ganska bra | | - Fair | |
| - Dålig | | - Bad | |
| - Väldigt dålig | | - Very bad | |
| - Jag vet inte/jag vill inte svara | | - Do not know/ do not want to answer | |
| Under de senaste 2 åren, har du varit i kontakt med läkare (primärvård eller specialist) eller annan vårdpersonal för: | | Have you been in contact with a HCP in the last two years for any of the following: | |
| *Markera alla svar som stämmer för dig, flera val är möjligt.* | | *Choose all the options that apply to you, multiple choices are possible.* | |
| - Psykisk ohälsa | | - Mental health | |
| - Cancer | | - Cancer | |
| - Andra hälsoproblem | | - Other health problems | |
| - Jag har inte fått någon vård/behandling | | - I have not received any care/ treatment | |
| Hur du läst information om din vård för psykisk ohälsa online i tjänsten Journalen? | | Have you read about your mental healthcare online in Journalen? | |
| - Jag har läst allt/nästan allt i Journalen | | - I have read all/almost all records in Journalen | |
| - Jag har läst delar av Journalen | | - I have read some of the records in Journalen | |
| - Jag har inte läst i Journalen | | - I did not read the record in Journalen | |
| När du fick vård för psykisk ohälsa, på vilken vårdnivå ägde detta rum? | | When you received mental healthcare, at what level of care did it take place? | |
| *Markera alla svar som stämmer för dig, flera val är möjligt.* | | *Choose all the options that apply to you, multiple choices are possible.* | |
| - Primärvård (vårdcentral) | | - Primary care | |
| - Psykiatri öppenvård (specialistkonsultation, ej inlagd) | | - Psychiatry outpatient (specialist consultation, not hospitalised)) | |
| - Psykiatri slutenvård (inlagd) | | - Psychiatry inpatient (hospitalised) | |
| - Akutsjukvård | | - Emergency care | |
| Hur länge fått vård för psykisk ohälsa? | | For how long have you received mental healthcare? | |
| - Mindre än 3 månader | | - Less than 3 months | |
| - 3 månader - 1 år | | - 3 months to 1 year | |
| - 1-3 år | | - 1 to 3 years | |
| - Längre än 3 år | | - More than 3 years | |
| **EXPERIENCE WITH ONLINE RECORD ACCESS THROUGH PATIENT PORTAL** | | | |
| Hur ofta har du läst din journal under senaste 12 månaderna? | | How often have you read your EHR during the last 12 months? | |
| - Det här är första gången | | - This is my first time | |
| - 2-9 gånger | | - 2 to 9 times | |
| - 10-20 gånger | | - 10 to 20 times | |
| - Mer än 20 gånger | | - More than 20 times | |
| Har du haft någon särskild positiv upplevelse av Journalen? | | Have you had a very positive experience with Journalen? | |
| - Ja | | - Yes | |
| - Nej | | - No | |
| Har du haft någon särskild negativ upplevelse av Journalen? | | Have you had a very negative experience with Journalen? | |
| - Ja | | - Yes | |
| - Nej | | - No | |
| Har du blivit uppmuntrad eller påmind att läsa din journal av någon/något av följande: | | Did any of the following encourage or remind you to read your health record: | |
| *Markera alla svar som stämmer för dig, flera val är möjligt.* | | *Choose all the options that apply to you, multiple choices are possible.* | |
| - Vårdpersonal *(om Ja: uppföljning om vilken vårdprofession)* | | - Healthcare professional *(if Yes: follow-up on what profession)* | |
| - Skriftlig information från sjukhuset/vårdinrättning/klinik | | - Written information from the hospital/care unit/clinic | |
| - Familj eller vänner | | - Family or friends | |
| - Webbsidor, såsom 1177.se Vårdguiden, etc. | | - Web-pages, such as 1177.se Vårdguiden, etc. | |
| - Tidningar, radio, TV, Facebook, etc. | | - Newspaper, radio, TV, Facebook, etc. | |
| - Andra patienter | | - Other patients | |
| - Ingen har uppmuntrat eller påmint mig | | - Nobody have encouraged or reminded me | |
| - Annat | | - Other | |
| **EXPERIENCE WITH ONLINE RECORD ACCESS THROUGH PATIENT PORTAL** | | | |
| Markera i vilken utsträckning du instämmer med följande påståenden: | | Mark the extent to which you agree with the following statements: | |
| *Att ha tillgång till min journal…* | | *Having access to my EHR…* | |
| Ökar min tillit till mina vårdgivare | | Helps me trust my care provider more | |
| 1. Instämmer inte 2 3 4 5. Instämmer | | 1. Disagree 2 3 4 5. Agree | |
| Stödjer bättre kommunikation mellan mig och vårdpersonal | | Supports communication between myself and my care provider | |
| 1. Instämmer inte 2 3 4 5. Instämmer | | 1. Disagree 2 3 4 5. Agree | |
| **ERRORS, OMISSIONS, & OFFENCE** | | | |
| Har du någonsin stött på felaktigheter i din journal (inte inräknat stavfel eller typografiska fel)? | | Have you ever found anything in your EHR you thought was a mistake (not misspellings or typographical errors)? | |
| - Ja | | - Yes | |
| - Nej | | - No | |
| - Vet inte/kommer inte ihåg | | - Do not know /do not remember | |
| Hur viktig var den mest allvarliga felaktigheten för dig? | | How important was the most serious mistake you found? | |
| - Inte alls viktig | | - Not at all important | |
| - Något viktig | | - Somewhat important | |
| - Väldigt viktig | | - Very important | |
| - Jag är osäker | | - I am not sure | |
| Har du någonsin stött på att viktig information saknas i din journal? | | Have you ever found anything in your EHR you thought was missing? | |
| - Ja | | - Yes | |
| - Nej | | - No | |
| - Vet inte/kommer inte ihåg | | - Do not know /do not remember | |
| Hur viktig var den mest allvarliga saknade informationen för dig? | | How serious was the most important missing information you found? | |
| - Inte alls viktig | | - Not at all important | |
| - Något viktig | | - Somewhat important | |
| - Väldigt viktig | | - Very important | |
| - Jag är osäker | | - I am not sure | |
| Gjorde du något av följande när du stötte på felaktigheter eller saknad information i din journal? | | Did you do any of the following when you found a mistake or missing information in your EHR? | |
| *Välj det viktigaste alternativet.* | | *Choose the most important option.* | |
| - Informerade vårdpersonalen vid nästa besök | | - Informed the healthcare professional at the next visit | |
| - Kontaktade vårdinrättningen över telefon | | - Contacted the care unit via phone | |
| - Gjorde inte något | | - Did nothing | |
| - Gjorde något annat | | - Something else | |
| Hur lätt (eller svårt) är det för dig att hitta felaktigheter i din journal? | | How easy (or difficult) is it for you to notice mistakes or errors in your EHR? | |
| 1. Väldigt svårt 2 3 4 5. Väldigt lätt | | 1. Very difficult 2 3 4 5. Very easy | |
| Har du någonsin känt dig förolämpad/sårad/illa berörd av något du läst i din journal? | | Have you ever felt offended by something you read in your EHR? | |
| - Ja | | - Yes | |
| - Nej | | - No | |
