# Supplementary material for: Errors, Omissions, and Offenses in the Health Record of Mental Health Care Patients: Results from a Nationwide Survey in Sweden
Source: J Med Internet Res. 2023 Nov 3;25:e47841. doi: 10.2196/47841 (PMC10656659; doi:10.2196/47841)
Supplement: Multimedia Appendix 2 [file jmir_v25i1e47841_app2.docx]

**Multimedia Appendix 2**. Checklist for Reporting Results of Internet E-Surveys (CHERRIES).

| ***Item Category*** | ***Checklist item*** | ***Described in the manuscript*** | ***Notes*** |
| --- | --- | --- | --- |
| **Design** | Describe survey design | Yes |  |
| **IRB (Institutional Review Board) approval and informed consent process** | IRB approval | Yes |  |
|  | Informed consent | Yes |  |
|  | Data protection | Yes |  |
| **Development and pre-testing** | Development and testing | Yes |  |
| **Recruitment process and description of the sample having access to the questionnaire** | Open survey versus closed survey | No | Open survey |
|  | Contact mode | Yes |  |
|  | Advertising the survey | Yes |  |
| **Survey administration** | Web/E-mail | Yes |  |
|  | Context | Yes |  |
|  | Mandatory/voluntary | Yes |  |
|  | Incentives | Yes |  |
|  | Time/Date | Yes |  |
|  | Randomization of items or questionnaires | No | Survey items were not randomized. |
|  | Adaptive questioning | No | Adaptive questioning was used for certain items. |
|  | Number of Items | No | 83 items in total. |
|  | Number of screens (pages) | No | 12 pages |
|  | Completeness check | No | No completeness check |
|  | Review step | No | Yes |
| **Response rates** | Unique site visitor | No | We did not determine unique site visitors. |
|  | View rate (Ratio of unique survey visitors/unique site visitors) | No | No view rates. |
|  | Participation rate (Ratio of unique visitors who agreed to participate/unique first survey page visitors) | No | 23878 clicked on the survey link. |
|  | Completion rate (Ratio of users who finished the survey/users who agreed to participate) | No | 13008 respondents completed the survey. |
| **Preventing multiple entries from the same individual** | Cookies used | No | No cookies used. |
|  | IP check | No | No IP check. |
|  | Log file analysis | No | No log file analysis. |
|  | Registration | No | The respondent had to login to a national PAEHR (Journalen) to get access to the survey link, which was an open survey. However, the survey participants could only respond to the survey once. |
| **Analysis** | Handling of incomplete questionnaires | No | Only fully completed questionnaires were analyzed. |
|  | Questionnaires submitted with an atypical timestamp | No | Time was not measured. |
|  | Statistical correction | No | Only participants aged 15 years or above were eligible to take part. |
