# Supplementary material for: Errors, Omissions, and Offenses in the Health Record of Mental Health Care Patients: Results from a Nationwide Survey in Sweden
Source: J Med Internet Res. 2023 Nov 3;25:e47841. doi: 10.2196/47841 (PMC10656659; doi:10.2196/47841)
Supplement: Multimedia Appendix 3 [file jmir_v25i1e47841_app3.docx]

**Multimedia Appendix 3**. Strata of the stratified random sampling.

| **Age** | **Gender** | **MH Original** | **non-MH original** | **MH Target** | **non-MH Target** | **TOTAL** |
| --- | --- | --- | --- | --- | --- | --- |
| 15-19 | Man | 15 | 16 | 15 | 15 * | 30 |
|  | Woman | 95 ** | 57 | 57 * | 57 | 114 |
| 20-24 | Man | 39 ** | 23 | 23 * | 23 | 46 |
|  | Woman | 190 ** | 124 | 124 * | 124 | 248 |
| 25-34 | Man | 139 ** | 115 | 115 * | 115 | 230 |
|  | Woman | 659 ** | 644 | 644 * | 644 | 1288 |
| 35-44 | Man | 129 | 216 | 129 | 129 * | 258 |
|  | Woman | 518 | 832 | 518 | 518 * | 1036 |
| 45-54 | Man | 164 | 425 | 164 | 164 * | 328 |
|  | Woman | 470 | 1095 | 470 | 470 * | 940 |
| 55-64 | Man | 141 | 647 | 141 | 141 * | 282 |
|  | Woman | 324 | 1323 | 324 | 324 * | 648 |
| 65-74 | Man | 50 | 1057 | 50 | 50 * | 100 |
|  | Woman | 91 | 1344 | 91 | 91 * | 182 |
| 75-84 | Man | 22 | 698 | 22 | 22 * | 44 |
|  | Woman | 26 | 483 | 26 | 26* | 52 |
| 85 > | Man | 2 | 51 | 2 | 2 * | 4 |
|  | Woman | 0 | 34 | 0 | 0 * | 0 |
| **TOTAL** | | 3074 | 9184 | 2915 | 2915 |  |

* Need to stratify

** Not enough data
